# Vitronectin, a Novel Urinary Proteomic Biomarker, Promotes Cell Pyroptosis in Juvenile Systemic Lupus Erythematosus

**DOI:** 10.1155/2022/8447675

**Published:** 2022-04-13

**Authors:** Song Zhang, Wenxu Pan, Hongli Wang, Cheng Zhi, Yanhao Lin, Ping Wu, Qi Ren, Ping Wei, Rui Chen, Feng Li, Ying Xie, Chun Kwok Wong, Hong Tang, Zhe Cai, Wanfu Xu, Huasong Zeng

**Affiliations:** ^1^The First Affiliated Hospital of Jinan University, Jinan University, Guangzhou, China; ^2^Department of Allergy, Immunology and Rheumatology, Guangzhou Women and Children's Medical Center, Guangzhou 510623, China; ^3^Department of Gastroenterology, Guangzhou Women and Children's Medical Center, Guangzhou Medical University, Guangzhou 510623, China; ^4^Department of Pathology, The Second Affiliated Hospital of Guangzhou Medical University, Guangzhou 510260, China; ^5^Heyuan Heping County Maternal and Child Health Hospital, Heyuan, Guangdong 517200, China; ^6^Shenzhen Bionavi Life Sciences Co., Ltd., Shenzhen, Guangdong 518118, China; ^7^Department of Chemical Pathology, The Chinese University of Hong Kong, Prince of Wales Hospital, Hong Kong 999077, China; ^8^Institute of Chinese Medicine and State Key Laboratory of Research on Bioactivities and Clinical Applications of Medicinal Plants, The Chinese University of Hong Kong, Hong Kong, China; ^9^Institute Pasteur of Shanghai, Chinese Academy of Science, Shanghai 200031, China; ^10^Guangzhou Institute of Pediatrics, Guangzhou Women and Children's Medical Center, Guangzhou Medical University, Guangzhou 510623, China

## Abstract

**Objective:**

Identifying new markers of juvenile systemic lupus erythematosus (JSLE) is critical event to predict patient stratification and prognosis. The aim of the present study is to analyze alteration of urinary protein expression and screen potential valuable biomarkers in juvenile systemic lupus erythematosus (JSLE).

**Methods:**

The urine was collected from the patients with or without JSLE and detected by mass spectrometry to analyze proteomic changes. ELISA was used to verify the Vitronectin (VTN) changes in a new set of patients. The clinical correlation was performed to analyze between VTN and clinical pathological parameters. WB and ELISA were used to analyze VTN-mediated cell pyroptosis.

**Results:**

Herein, we have identified a group of 105 differentially expressed proteins with ≥1.3-fold upregulation or ≤0.77-fold downregulation in JSLE patients. These proteins were involved in several important biological processes, including acute phase inflammatory responses, complement activation, hemostasis, and immune system regulation through Gene Ontology and functional enrichment analysis. Interestingly, urinary ephrin type-A receptor 4 (EPHA4) and VTN were significantly reduced in both inactive and active JSLE patients, and VTN treatment in THP-1 derived macrophages led to a significant increased cell pyroptosis by activation of Nod-like receptor family protein 3 (NLRP3) inflammasomes, resulting in caspase-1 activation, cleaved gasdermin D (GSDMD), and IL-18 secretion. Most importantly, the urinary VTN was also linearly correlated with clinical characteristics of JSLE, implying that VTN could be a specific diagnostic biomarker to distinguish inactive and active JSLE.

**Conclusion:**

This study provided a novel role of VTN in pyroptosis in JSLE through the urinary proteomic profile for JSLE, which could be a nonintrusive monitoring strategy in clinical diagnosis.

## 1. Introduction

Systemic lupus erythematosus (SLE), an autoimmune disease with inflammation, affects multiple organ systems characterized by excessive production of antinuclear antibodies and high morbidity and low quality of life [1–3]. The precise pathophysiological mechanism of SLE is largely unknown. One of the most important mechanisms involved in SLE is alteration of immune reactions, which involved autoantibodies that target the patient's own tissues and subsequently lead to the inflammation [4]. Despite tremendous basic and clinical research progress regarding treatment [5], various cytokines and chemokines have emerged as the potential biomarkers of SLE disease activity [6]. However, due to the complexity of this disease, the clinical usefulness of these potential biomarkers in assessing disease in SLE is not well established, especially through noninvasive and easy collected urine samples to diagnose renal activity of SLE.

The changes in humoral metabolomics, including serum, plasma, urine, and sputum, can provide abundant information on the physiological and pathological states of individuals and useful clinical parameters. Consequently, to understand the enormous potential in terms of revealing disease conditions will also bring the promise of a revolution in disease diagnosis and therapeutic monitoring [7–10]. In a previous study, 23 differential metabolites and 5 perturbed pathways including aminoacyl-tRNA biosynthesis, thiamine metabolism, nitrogen metabolism, tryptophan metabolism, and cyanoamino acid metabolism were identified between SLE patients and healthy controls [1]. Also, the studies exhibited that immunoglobulin-binding protein [7], urinary vitamin D-binding protein, and S100 calcium-binding protein (S100) were the potential biomarkers in patients with lupus nephritis [11, 12]. With significant advances in proteomic technologies, the comprehensive profiling of protein expression in biofluids from patients with a given disease prompts a deep exploration of disease and its underlying mechanisms. In an attempt to reach greater understanding of the pathogenesis, based on advantage of proteomic technologies, we tried to seek potential information in urine to differentiate inactive juvenile SLE (JSLE) patients from active ones and healthy donors.

Vitronectin (VTN), a multifunctional glycoprotein, has been demonstrated to be enriched in the serum, extracellular matrix, and platelets [13] and regulated cell adhesion, coagulation, fibrinolysis, complement activation, and apoptosis by interacting with integrin av*β*3, plasminogen activator Inhibitor-1 (PAI-1), and urokinase plasminogen activator (uPAR) [14, 15]. Also, it has been reported that VTN was associated with inflammation in several biological processes, such as acute lung injury, burns, and sepsis, which were involved in the neutrophil and autoreactive T and B lymphocyte activation and tissue injury [16–19]. In addition to VTN, ephrin type-A receptor 4 (EPHA4), belonging to the ephrin receptor subfamily of the protein-tyrosine kinase family, has been implicated in mediating developmental events and associated with the development of SLE, particularly in the central nervous system lupus erythematosus (CNS-SLE) [20, 21]. What is more, ephrin-A1-EphA4 signaling has reported to negatively regulate with myelination in the central nerve system [20]. Up to now, some proteomic changes of urine have been described in SLE patients. However, the proteomics of urinary specific biomarkers that distinguished the inactive JSLE and active JSLE remained to be identified.

In this study, we further showed that the VTN and EPHA4, decreased in JSLE by urinary protein profiling, could be considered the novel biomarkers associated with the autoimmune inflammation among healthy donors and inactive and active JSLE patients. Therefore, the alteration of VTN and EPHA4 proteins could be a potential strategy to develop innovative therapeutic approaches.

## 2. Materials and Methods

### 2.1. Patients and Study Design

A total of 9 healthy controls (group 1/G1) and 19 patients diagnosed with SLE (group 2/G2: 9 inactive JSLE patients; group 3/G3: 10 active JSLE patients) were recruited in Guangzhou Women and Children's Medical Center. The written informed consent was obtained from all subjects according to the Declaration of Helsinki. This clinical ethical was approved by the Ethics Committee of Guangzhou Women and Children's Medical Center. The clean morning urine samples were collected and centrifuged at 3000 g, at 4°C for 15 min. The supernatant was stored at -80°C for further proteomics analysis.

### 2.2. Disease Activity Assessment

Disease activity assessment was performed using the modified SLE Disease Activity Index 2000 (SLEDAI-2K) described in the previous study [22]. The modified SLEDAI-2K excludes 2 immunological items (the complement levels and anti-double-stranded DNA/dsDNA) from the original SLEDAI-2K, while maintaining the 16 clinical manifestations and 4 laboratory tests (white blood cell count, platelet count, urinalysis, and 24-hour proteinuria). Since the British Isles Lupus Assessment Group 2004 (BILAG-2004) index provided detailed assessments including severity for affected organs and system manifestations [23], we have modified the total BILAG-2004 index based on the scorings of *A* = 12, *B* = 8, *C* = 1, and *D*/*E* = 0. The inactive disease of JSLE patients was defined as above modified BILAG-2004 index *C*/*D*/*E* or modified SLEDAI-2K scores < 5. The active JSLE patients were determined by modified BILAG-2004 index *A*/*B* or modified SLEDAI-2K scores ≥ 5. Several clinical manifestations of healthy controls were also demonstrated in the comparison to JSLE patients. Therefore, the correlation between the differentiated protein levels and the severity of certain organ/system manifestations of JSLE patients were also determined.

### 2.3. Sample Preparation, Mass Spectrometry (MS), and MS Interpretation

All samples were prepared for proteomics assay with the following preparations. Protein concentration in the urine was determined using a BCA kit (Beyotime, P0009) according to the manufacturer's instructions. For digestion, dithiothreitol was used to concentrate the total protein solution for 30 min at 56°C, and then, alkylation of protein with 11 mmol/L iodoacetamide was performed for 15 min at room temperature in the dark. After trypsin digestion, the peptides were combined and dried and then subjected to an nitrogen solubility index source followed by tandem mass spectrometry (MS/MS) in Q-Exactive (Thermo Fisher Scientific, San Jose, CA, USA) coupled online to an ultraperformance liquid chromatography system. The resulting MS/MS data were processed using MaxQuant with the integrated Andromeda search engine (v.1.5.2.8). Gene Ontology (GO) was a major bioinformatics initiative to unify the representation of the gene and gene product attributes across all species. The GO annotation proteome was derived from the UniProt-GOA database (http://www.ebi.ac.uk/GOA). The Kyoto Encyclopedia of Genes and Genomes (KEGG) was used to connect known information on molecular interaction networks. These pathways were classified into hierarchical categories according to the KEGG website.

### 2.4. Protein-Protein Interaction (PPI) Network Construction and Module Analysis

The Search Tool for the Retrieval of Interacting Genes (STRING) online database (https://string-db.org/cgi/input.pl) tool was employed to construct the PPI network.

### 2.5. Cell Culture and Treatment

THP-1 cells were purchased from American Type Culture Collection (ATCC, Manassas, VA) and cultured according to the manufacturer's recommendations. Dulbecco's modified Eagle medium (DMEM) and fetal bovine serum (FBS) were purchased from Gibco (Thermo Fisher Scientific, USA). The cells were treated with PMA (100 nM, P6741, Solarbio, Beijing, China) for 72 hours and induced into macrophages. For cell treatment, VTN (P00082, Solarbio, Beijing, China) was used to treat THP-1-derived macrophages at 4 *μ*M. Other reagents used in this study were purchased from Sigma.

### 2.6. Western Blotting

As described in our previous study [24], the whole cell lysate was harvested and subjected to SDS-PAGE and transferred into the nitrocellulose transfer membrane. After incubation with 5% (*w*/*v*) milk in PBS/0.05% (*v*/*v*) Tween-20 for 1 hour, the membrane was incubated with indicated antibodies overnight at 4°C, subsequently followed by incubation with a horseradish peroxidase secondary antibody (Jackson ImmunoResearch) for 1 hour at room temperature. Proteins were detected using an enhanced chemiluminescence (PerkinElmer). The antibodies used in this study were from Abcam: NLRP3 (ab263899, 1 : 2000 for WB), IL-1*β* (ab254360, 1 : 1000 for WB), caspase-1 (ab179515, 1 : 1000 for WB), ASC (AB283684,1 : 2000 for WB), GSDMD (ab219800, 1 : 2000 for WB), NF-*κ*B (ab207297, 1 : 2000 for WB), phospho-NF-*κ*B (ab239882, 1 : 2000 for WB), *β*-actin (ab8226, 1 : 4000 for WB), and *α*-tubulin (ab7291, 1 : 5000 for WB).

### 2.7. Real-Time PCR

As described in our previous study [25], the total RNA was extracted according to the instruction. The All-in-One First-Strand cDNA Synthesis Kit and All-in-One qPCR Mix were used to perform reverse transcription and quantitative PCR (qPCR) according to the manufacturer's protocol. The primers used in this study are as follows: IL-1*β*: forward: 5′-ATGATGGCTTATTACAGTGGCAA-3′ and reverse: 5′-GTCGGAGATTCGTAGCTGGA-3′; IL-18: forward: 5′-TCTTCATTGACCAAGGAAATCGG-3′ and reverse: 5′-TCCGGGGTGCATTATCTCTAC-3′; GSDMD: forward: 5′-GTGTGTCAACCTGTCTATCAAGG-3′ and reverse: 5′-CATGGCATCGTAGAAGTGGAAG-3′; NLRP3: forward: 5′-GATCTTCGCTGCGATCAACAG-3′ and reverse: 5′-CGTGCATTATCTGAACCCCAC-3′; ASC: forward: 5′-TGGATGCTCTGTACGGGAAG-3′ and reverse: 5′-CCAGGCTGGTGTGAAACTGAA-3′; caspase-1: forward: 5′-CCTTAATATGCAAGACTCTCAAGGA-3′ and reverse: 5′-TAAGCTGGGTTGTCCTGCACT-3′; and UBC: forward: 5′-ATTTGGGTCGCGGTTCTTG-3′ and reverse: 5′-TGCCTTGACATTCTCGATGGT-3′.

### 2.8. ELISA

IL-1*β* (ab229384) and IL-18 (ab215539) in culture supernatants were measured and quantitated for the indicated group by ELISA according to the manufacturer's instructions, respectively.

### 2.9. Relative Cell Death Assays

LDH assay kit (Abcam, ab102526) was used to analyze LDH in supernatants from THP-1-derived macrophages treated in the experiment according to the manufacturer's instructions. Relative cell death was determined as described in Zhou et al.'s study [26].

### 2.10. Statistical Analysis

Statistical analysis of *in vivo* data was described in the section of each assay. Results were expressed as mean ± standard deviation (SD) for normally distributed data. Mann-Whitney *U* tests were tested for the continuous variables. Comparison of different groups was made with Kruskal-Wallis analysis of variance (ANOVA), followed by Dunn's posttest for comparing the differences and calculating a probability (*p*) value for each pair of comparison. All hypotheses were two-tailed, and *p* values less than 0.05 were considered significant. Spearman's rank correlation test was used to assess the lineal correlations among the urinary VTN expression with clinical parameters of JSLE patients. *r* is the correlation coefficient. The *t*-test was performed in qPCR and ELISA analysis. Data were analyzed using GraphPad Prism (GraphPad Software, La Jolla, CA, USA).

## 3. Results

### 3.1. Patients and Clinical Characteristics

As shown in Table 1, a total of 28 early morning urine samples were collected from 9 healthy controls, 9 patients with inactive JSLE, and 10 patients with active JSLE. We found that creatinine level in healthy control is lower than inactive JSLE patients (22.87 ± 4.37 versus 61.13 ± 15.99, *p* < 0.05); the higher mean ratio of aspartate amino transferase (AST)/alanine amino transferase (ALT) was observed in inactive (1.47 ± 0.62 versus 2.54 ± 0.79, *p* < 0.01) and active (1.36 ± 0.72 versus 2.54 ± 0.79, *p* < 0.01) JSLE patients compared with healthy controls, respectively, while lower hemoglobin (HB) levels in active JSLE patients (96.78 ± 25.92 g/L) were found compared to those in healthy controls (96.78 ± 25.92 versus 126.10 ± 31.90) and inactive JSLE patients (96.78 ± 25.92 versus 116.00 ± 21.33), respectively.

In comparison with inactive JSLE patients, SLEDAI (11.50 ± 7.29 versus 4.11 ± 4.08, *p* < 0.05), ANA (417.60 ± 201.80 versus 16.71 ± 14.85, *p* < 0.001), and dsDNA (443.10 ± 309.70 versus 14.52 ± 12.27, *p* < 0.01) were higher, while erythrocyte sedimentation rate (ESR) (6.50 ± 6.76 versus 29.11 ± 22.86, *p* < 0.05) and complement C3 (0.38 ± 0.39 versus 0.75 ± 0.13, *p* < 0.05) and C4 (0.06 ± 0.05 versus 0.14 ± 0.04, *p* < 0.01) in active JSLE patients. However, no statistical differences were obtained in analysis of SLICC, proteinuria, white blood cell (WBC), blood platelet (PLT), and C-reactive protein (CRP). The detailed statistical differences in clinical characteristics between JSLE patients and healthy controls are listed in Table 1.

### 3.2. Prolife of the Proteome in the Urine of JSLE Patients

**Figure 1:**
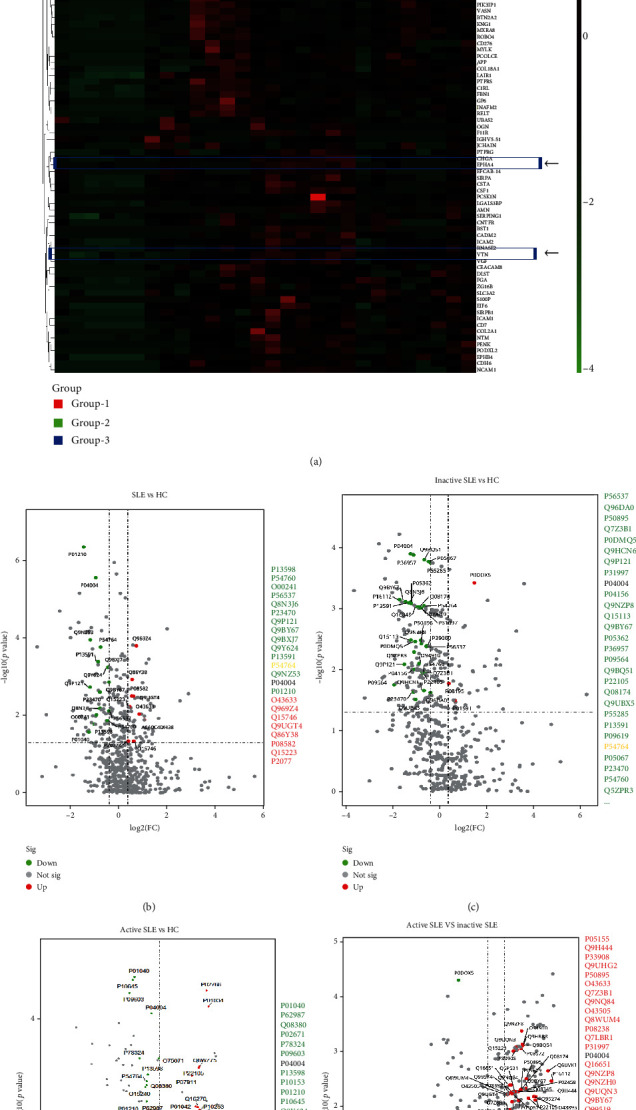
Profile of urinary proteins. (a) Heatmap shows the differentially expressed genes (DEGs) reflected by differentially expressed matching proteins in the indicated groups (group 1/G1: healthy controls, group 2/G2: inactive JSLE patients, and group 3/G3: active JSLE patients). Red and green colors indicate higher and lower expression, respectively. (b–e) Volcano dot plot presents the mass spectrometry data of proteome as -log10 (*p* value) plotted against the log2 (fold change/FC). The -log10 (*p* value) and log2 (FC) analysis thresholds are indicated by dotted lines (≥1.3-fold upregulation or ≤0.77-fold downregulation, *p* < 0.05). Green dots represent the downregulated proteins, while red dots represent the upregulated proteins and gray dots represent no significant difference in proteins. The symbol of differentially expressed proteins labeled with relevant color is listed beside each graph.

Proteomics analysis was then performed on the urine samples. As shown in Figure1(a), heatmap results showed that 105 different proteins have been identified among groups (group 1/G1: healthy controls; group 2/G2: inactive JSLE patients; and group3/G3: active JSLE patients) through liquid chromatography- (LC-) mass spectrometry (MS). Bioinformatics analysis of 16 downregulated proteins (≤0.77-fold downregulation) and 9 upregulated proteins (≥1.3-fold upregulation) was obtained between JSLE patients (G2+G3) and healthy controls (Figures 1(a) and 1(b)). Further results showed that a total of 3 increased (≥1.3-fold upregulation) and 34 decreased (≤0.77-fold downregulation) proteins were quantified between G2 and G1 (Figure 1(c)), while 21 increased (≥1.3-fold upregulation) and 16 decreased (≤0.77 downregulation) proteins were observed in a comparison between G3 and G1 (Figure1(d)). Interestingly, compared to the G2, only 2 decreased proteins and 47 increased proteins were found in the urine of active JSLE patients (Figure 1(e)).

**Figure 2:**
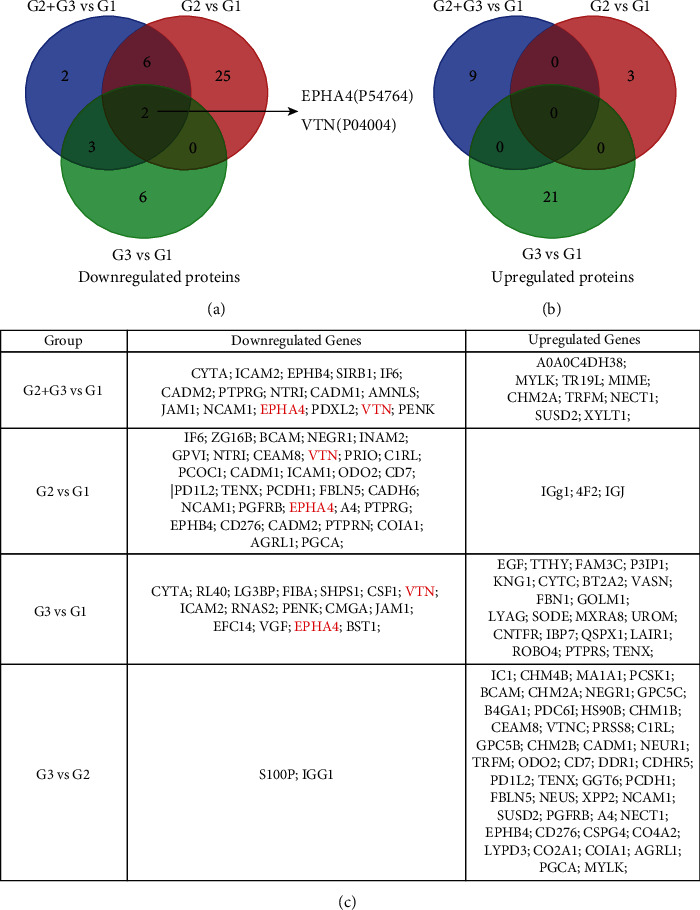
Identification of aberrant differentially expressed proteins including downregulated and upregulated proteins. (a) Two aberrantly downregulated proteins EPHA4 and VTN, (b) but no aberrantly upregulated proteins are obtained by Venn diagram among the indicated groups (G1: healthy controls, G2: inactive JSLE patients, and G3: active JSLE patients). (c) The aberrant DEGs reflected by their matching proteins are listed in the indicated groups.

Based on these findings, to further explore the specific differentially expressed genes (DEGs) among three groups, we overlapped the downregulated proteins that reflected gene expressions, and we achieved two downregulated proteins in JSLE patients compared with healthy controls (VTN and EPHA4, shown in Figure 2(a)), whereas none was obtained by overlapping the upregulated proteins (Figure 2(b)). All these DEGs are listed in Figure 2(c).

### 3.3. Functional Enrichment Analysis

To further analyze these DEGs involved in the possible biological function, the enrichment analysis was performed by the GO and KEGG terms, including molecular function, cellular component, and biological process that were significantly enriched to indicate the nature of the differentially expressed proteins in urine between JSLE patients and healthy controls using the Database for Annotation, Visualization and Integrated Discovery online tool.

**Figure 3:**
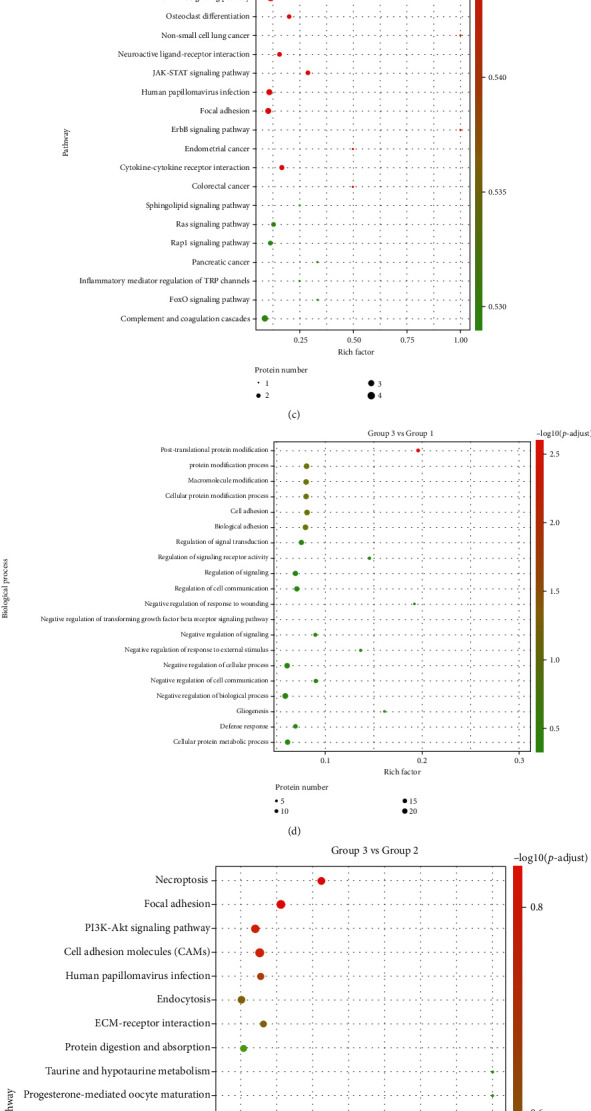
Functional enrichment analysis of signaling pathways and biological process of DEGs. A comparison of top 20 Gene Ontology terms of signaling pathways (a, c, e) and biological process (b, d, f) is indicated among groups (group 1: healthy controls, group 2: inactive JSLE patients, and group 3: active JSLE patients).

As for the DEGs in analysis of inactive JSLE patients and healthy controls, multiple enriched GO terms and KEGG pathways were demonstrated. The top 20 enriched GO terms and KEGG pathways were selected and are shown in Figure 3.  Figure 3(b) showed that the main enrich GO terms were majorly associated with cell adhesion, biological adhesion, peptidyl-tyrosine phosphorylation, peptidyl-tyrosine modification, and cell-cell adhesion via plasma-membrane adhesion molecules. When compared with healthy controls and inactive JSLE patients, the differentiated KEGG pathways are listed in Figure 3(a). Also, the involvement of salivary secretion, the PI3K-Akt signaling pathway and human papillomavirus infection as well as focal adhesion were observed, while biological function was related to posttranslational protein modification, protein modification, cell adhesion, and cellular protein modification process in the comparison of active JSLE patients and healthy controls (Figures 3(c) and 3(d)). In addition, from the comparison results of inactive and active JSLE patients, we also found DEGs participating in multiple biological pathways, such as necroptosis, focal adhesion, and PI3K-Akt signaling that were involved in the regulation of mitotic nuclear division and nuclear division (Figures 3(e) and 3(f)).

### 3.4. The Protein-Protein Interaction Analysis of Differential Genes

**Figure 4:**
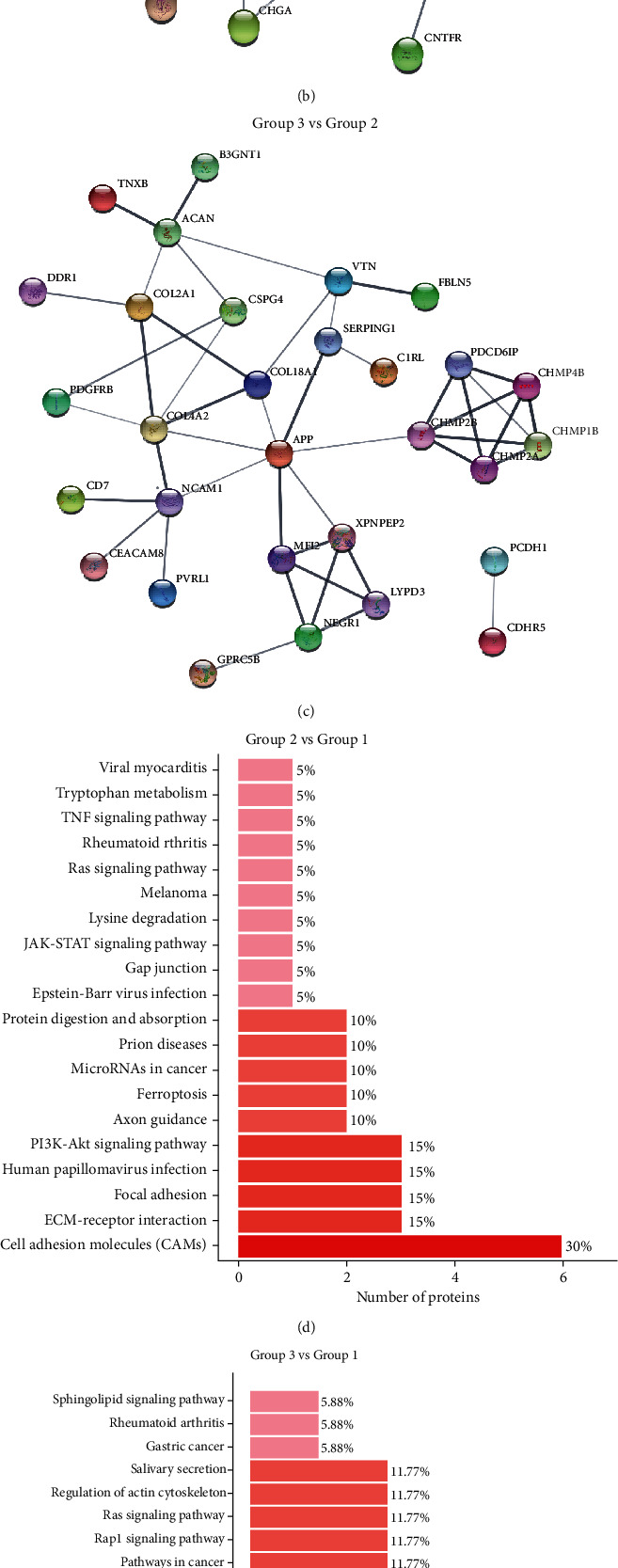
The protein-protein interaction (PPI) networks of common DEGs. (a–c) The comparisons of PPI networks of DEGs are shown among the indicated groups (group 1: healthy controls, group 2: inactive JSLE patients, and group 3: active JSLE patients) by using the STRING online database. The top 20 enrich KEGG pathways are selected and classified into hierarchical categories according to the comparison between groups.

To clearly understand the interaction of downregulated DEGs, the PPI network of these DEGs was generated to identify the key genes and their interactions in JSLE utilizing the STRING online database. As shown in Figure 4, these results showed that VTN is formed as a core molecule of the PPI network among three groups, at least interacting with other genes in the interatomic networks, including collagen type XVIII alpha 1 (COL18A1), fibulin 5 (FBLN5), aggrecan (ACAN), junctional adhesion molecule-A/F11 receptor (F11R), and prion protein (PRNP). As for the active and inactive JSLE, VTN, COL18A1, and ACAN were also significant hub genes in the interatomic networks.

### 3.5. Correlation between Urine VTN Levels and Lupus Clinical Parameters

**Figure 5:**
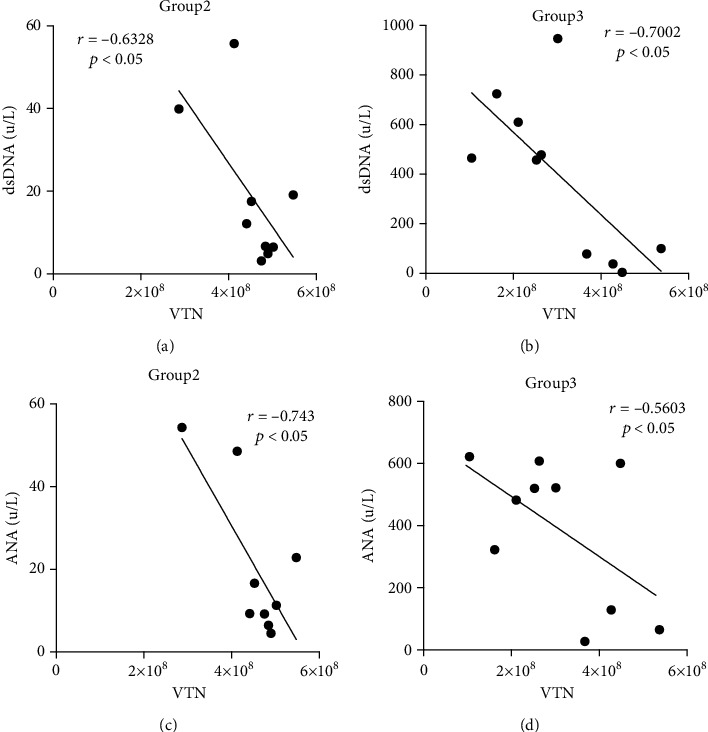
Clinical correlation between VTN with dsDNA, ANA, and complement C3 and C4. The dot plot shows the correlation between urinary VTN expression and (a) serum dsDNA level, (c) serum ANA level, (e) serum complement C3 level, and (g) serum complement C4 level in inactive JSLE patients, as well as the correlation between urinary VTN expression and (b) serum dsDNA level, (d) serum ANA level, (f) serum complement C3 level, and (h) serum complement C4 level in active JSLE patients (group 2: inactive JSLE patients, group 3: active JSLE patients).

In this study, upon the quantitative results, we tried to seek the clinical values for a detection of urinary VTN in JSLE patients. We found that VTN was negatively associated with serum dsDNA (inactive JSLE: *r* = −0.6328, *p* < 0.05, Figure 5(a); active JSLE: *r* = −0.7002, *p* < 0.05, Figure 5(b)), and the similar relationship was obtained in the correlation of ANA and VTN (inactive JSLE: *r* = −0.743, *p* < 0.05, Figure 5(c); active JSLE: *r* = −0.5603, *p* < 0.05, Figure 5(d)). In addition, complement C3 and C4 were positively associated with VTN in both inactive JSLE (C3: *r* = 0.6440, *p* < 0.05, Figure 5(e); C4: *r* = 0.8343, *p* < 0.01, Figure 5(g)) and active JSLE (C3: *r* = 0.7156, *p* < 0.05, Figure 5(f); C4: *r* = 0.8192, *p* < 0.01, Figure 5(h)).

### 3.6. Correlation between Urine VTN Levels and Different Lymphocyte Subsets

**Figure 6:**
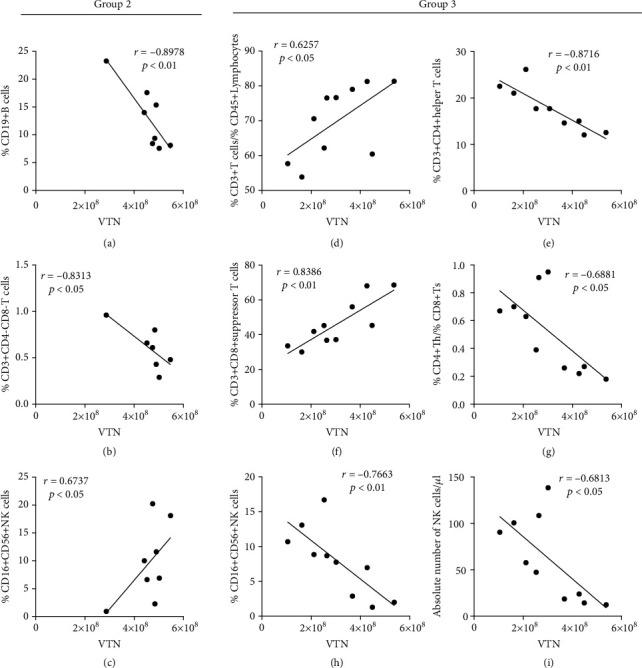
Clinical correlation between VTN and lymphocyte subsets. The dot plot shows the correlation between urinary VTN expression with (a) %CD19^+^ B cells, (c) % CD16^+^CD56^+^ NK cells in PBMC, and (b) %CD3^+^CD4-CD8- T cells in the whole blood of inactive JSLE patients, as well as the correlation between urinary VTN expression with (d) the ratio of %CD3^+^ T cells/%CD45^+^ lymphocytes, (e) %CD3^+^CD4^+^ helper T (Th) cells, (f) %CD3^+^CD8^+^ suppressor T (Ts) cells, (g) %CD4^+^ Th/%CD8^+^ Ts, (h) %CD16^+^CD56^+^ NK cells, and (i) absolute number of NK cells in PBMC of active JSLE patients (group 2: inactive JSLE patients, group 3: active JSLE patients).

SLE is characterized by the loss of tolerance to self-antigens, downstream activation, and expansion of autoreactive T and B lymphocytes [19]. In this study, upon the quantitative results, we found that urine VTN level was negatively associated with %CD19^+^ B cells (*r* = −0.8978, *p* < 0.01, Figure 6(a)) and %CD3^+^CD4^−^CD8^−^ T cells (*r* = −0.8313, *p* < 0.05, Figure 6(b)) in the peripheral blood mononuclear cell (PBMC), as well as positively associated with %CD16^+^CD56^+^ NK cells (*r* = 0.6724, *p* < 0.05, Figure 6(c)) in the whole blood of inactive JSLE. The similar relationship was obtained in an analysis for the correlations of VTN with the ratio of %CD3^+^ T cells/%CD45^+^ lymphocytes (*r* = 0.6257, *p* < 0.05, Figure 6(d)), %CD3^+^CD4^+^ helper T cells (Th) (*r* = −0.8716, *p* < 0.01, Figure 6(e)), and %CD3^+^CD8^+^ suppressor T cells (Ts) (*r* = 0.8385, *p* < 0.01, Figure 6(f)), the ratio of %CD4^+^ Th/%CD8^+^ Ts (*r* = −0.6881, *p* < 0.05, Figure 6(g)) and %CD16^+^CD56^+^ NK cells (*r* = −0.7663, *p* < 0.01, Figure 6(h)), and absolute number of NK cells (*r* = −0.6813, *p* < 0.01, Figure 6(i)) in PBMC of active JSLE. Moreover, no significant associations were observed between VTN and other index (data not shown).

### 3.7. VTN Induced Cell Pyroptosis in THP-1-Derived Macrophages

**Figure 7:**
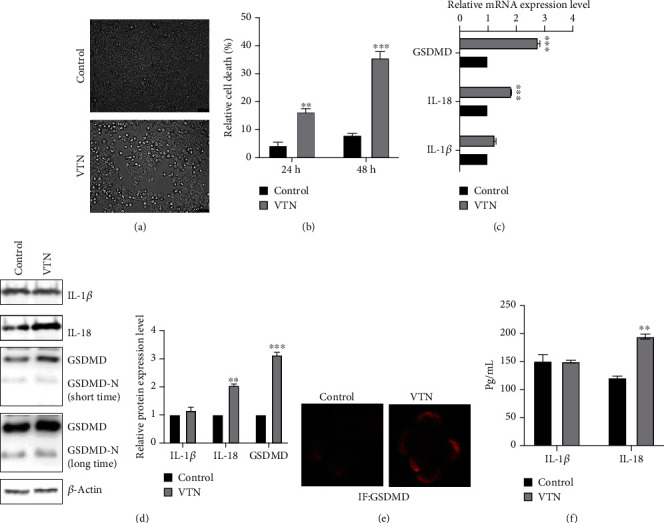
VTN triggered cell pyroptosis. (a) LDH assay was performed to determine relative cell death in the indicated group in THP-1-derived macrophages; data represented the mean ± SD of three independent experiments; *t*-tests were used to analyze statistical significance; ^∗∗∗^*p* < 0.001, ^∗∗^*p* < 0.01. (b) Real-time PCR was performed to examine the mRNA level of GSDMD, IL-1b, and IL-18 expression in THP-1-derived macrophages. Data represented the mean ± SD of three independent experiments; *t*-tests were used to analyze statistical significance; ^∗∗∗^*p* < 0.001. (c) Western blotting was performed to test pyroptosis-related proteins, including GSDMD, IL-1b, and IL-18, in THP-1-derived macrophages with or without VTN (4 *μ*M) stimulation, and the quantified results were analyzed by the *t*-test. Data represented the mean ± SD of three independent experiments; *t*-tests were used to analyze statistical significance; ^∗∗∗^*p* < 0.001. (d) The contents of IL-1b and IL-18 in the supernatant and GSDMD/GSDMD-N expression of THP-1-derived macrophages with or without VTN treatment were detected by ELISA; data represented the mean ± SD of three independent experiments; *t*-tests were used to analyze statistical significance; ^∗∗^*p* < 0.01. (e) IF assay was performed to detect GSDMD expression in THP-1-derived macrophages with or without VTN treatment.

To further explore the possible function of VTN in SLE, the THP-1-derived macrophages were used in the in vitro model and treated with or without VTN to detect cell pyroptosis change. As shown in Figure 7(a), morphologically, a larger number of dead cells were observed in THP-1-derived macrophages treated with VTN compared with the control group. The LDH release assay revealed that cell death was drastically increased in thp-1-derived macrophages in response to VTN stimulation (Figure 7(b)). These findings implied that VTN has a propyroptosis effect.

The results from the real-time PCR assay showed that VTN treatment led to a significant upregulation of IL-18 and GSDMD expression, a pivotal executioner of cell pyroptosis [27, 28], while no significant difference was obtained in IL-1*β* expression (Figure 7(c)). In line with this, the western blotting and quantified results also demonstrated that IL-18 and GSDMD expression was significantly enhanced in THP-1-derived macrophages treated by VTN stimulation compared with the control group (Figure 7(d)). Further results from ELISA suggested that IL-18 release was increased in response to VTN stimulation, and VTN has failed to alter IL-1*β* expression (Figure 7(e)). These findings suggested that VTN triggered GSDMD-executed cell pyroptosis, leading to the increase in IL-18 release to aggravate inflammation.

### 3.8. VTN Regulated Pyroptosis through Triggering NLRP3 Inflammasome Activation

**Figure 8:**
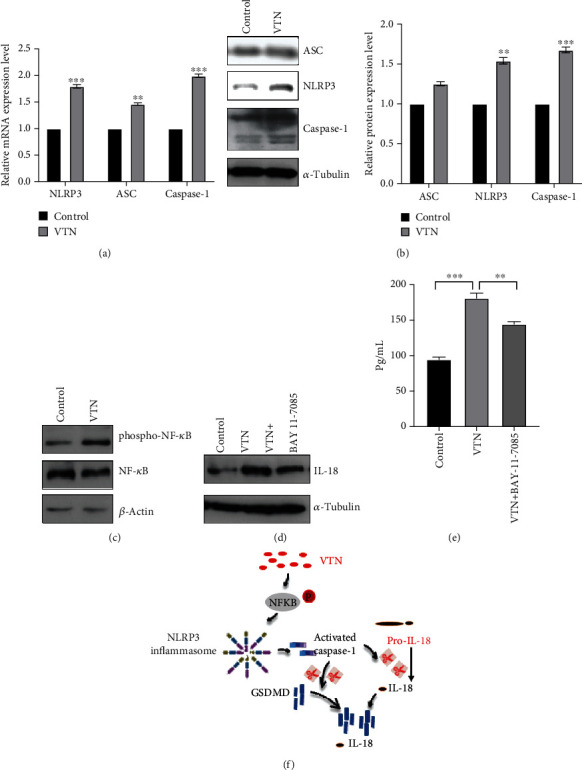
VTN triggered the NF-*κ*B pathway and NLRP3 inflammasome. (a) Real-time PCR and (b) western blotting were performed to detect ASC, NLRP3, and caspase-1 expression in THP-1-derived macrophages with or without VTN treatment. Data represented the mean ± SD of three independent experiments; *t*-tests were used to analyze statistical significance; ^∗^*p* < 0.05, ^∗∗^*p* < 0.01, and ^∗∗∗^*p* < 0.001. (c) Phosphorylation of NF-*κ*B was detected in THP-1-derived macrophages in response to VTN stimulation for 1 hour. (d, e) IL-18 was analyzed by western blotting and ELISA in THP-1-derived macrophages treated with VTN for 1 h, followed by BAY 11-7085 stimulation for another 48 hours. Data represented the mean ± SD of three independent experiments; one-ANOVA was used to analyze statistical significance; ^∗∗^*p* < 0.01, ^∗∗∗^*p* < 0.001. (f) The schematic illustration for VTN-regulated cell pyroptosis.

The NLRP3 inflammasome, the well-known characterized inflammasome, consists of NLRP3, apoptosis-associated speck-like protein containing a CARD (ASC), and caspase-1, which focused our attention to explore the effect of VTN on the NLRP3 inflammasome [29]. Based on this, we detected NLRP3 inflammasome changes in THP-1-derived macrophages with VTN stimulation. The results from qPCR and WB showed that VTN induced NLPR3 and caspase-1 expression in THP-1-derived macrophages, while no significant difference was obtained in ASC expression, leading to enhancing cleaved GSDMD and GSEMD-N expression (Figures 8(a) and 8(b)).

Our previous study has demonstrated that the NF-*κ*B pathway is involved in cell pyroptosis [24], which further asked us to reveal the potential mechanism underlying VTN-regulated cell pyroptosis. As shown in Figure 8(c), phosphorylation of NF-*κ*B was significantly increased in THP-1-derived macrophages in response to VTN treatment, while inhibition of NF-*κ*B by BAY 11-7085 could reverse the induced effect of VTN on IL-18 expression and secretion (Figures 8(d)–8(f)). These findings suggested that VTN promoted cell pyroptosis through the NF-*κ*B pathway.

## 4. Discussion

The development of SLE is closely associated with the alteration in molecular functions, biological processes, and signaling pathways. However, the molecular characteristics and the molecular functions, pathways, and interactions in different stages of SLE are not well understood. In the currently study, we revealed 105 different proteins in the comparison between JSLE patients and healthy controls based on the proteomic assay. The results showed that these specific DEGs had different molecular functions, biological pathways, and formed complex interactome networks. What is more, we have found both urine EPHA4 and VTN levels are reduced in inactive and active JSLE patients when compared to healthy controls. Further bioinformatics analysis implied us that the EPHA4 and VTN in urine might have a potential possibility to be biomarkers of JSLE patients because of the clinical relationship between the pathological parameter and VTN. Most importantly, VTN treatment led to a significant activation of NLRP3 inflammasomes, leading to cleaving GSDMD-N and IL-18 secretion to aggravate inflammation in THP-1-derived macrophages, suggesting that VTN might serve as a useful biomarker for clinical prognosis.

Coincidentally, it is reported that the high levels of VTN are associated with the circulating immune complex- (CIC-) soluble membrane attack complex (MAC) in SLE patients with active nephritis [30], implying the possible value of VTN in active nephritis. Interestingly, in our study, we further demonstrated a significant decreased level of urinary VTN and lower expression of serum C3 and C4 in inactive JSLE and also a positive association between VTN and C3/C4, which may derive from the metabolic dissociation of CIC-MAC in JSLE glomerulus, identifying that a novel mechanism may contribute to the JSLE nephritis. What is more, it has been demonstrated that EPHA4 performed an important role in a number of cellular processes, including promoting cell proliferation and cell adhesion-mediated drug resistance via the Akt signaling pathway [30], while COL18A1 interacted with VTN and EPHA4 to modulate acute liver injury through binding *α*1*β*1 integrin on hepatocytes [31]. In addition, EPHA4 receptor activation-mediated PI3K/Akt and Wnt/*β*-catenin signaling pathways as well as ERK1/2 play an important role in regulating epithelial-mesenchymal transition development [32], which is in line with the analysis of these pathways as shown in Figures 4(d) and 4(e), including PI3K-Akt, cell adhesion molecules, and focal adhesion. Specifically, EPHA4 is the most abundant ephrin receptor, which interacted with almost all ephrin ligands, ranging from effects on inflammatory responses to axonal degeneration and regeneration in the central nervous system, especially in neurological functional recovery by regulating various processes, such as neuroinflammation, angiogenesis, neurogenesis, axonal reorganization, and synaptic plasticity [33]. Although there is no available evidence that proved the function of EPHA4 in the pathogenesis of SLE, EPHA4 as a possible inflammatory mediator may contribute to the immunopathological disease like multiple sclerosis and neuropsychiatric disorder in SLE [34–39]. Occasionally, in our study, we have shown a significant decreased protein level of EPHA4 in the urine of JSLE patients, which implied a potential role of EPHA4 in neurodevelopment of JSLE patients via the PI3K-Akt signaling pathway. However, the correlation between EPHA4 and clinical parameters will be further addressed in the future study with a large number of JSLE patients.

In addition to the EPHA4, we found that dsDNA and ANA had a negative correlation with urine VTN level, which was similar with many other studies like circulating S100 and serum triggering receptor expressed on myeloid cell-1 [40, 41]. Otherwise, VTN was reported to involve in promoting the expression of inflammatory factors, such as IL-6 and leukemia inhibitory factor via integrin-focal adhesion kinase and uPAR signaling pathways [42], and to promote neurogenesis [43]. Another study also revealed an increased colocalization of MAC, VTN, and VTN receptor (av*β*3 integrin), both of which are within and around the subepithelial deposits in membranous nephropathy [44]. What is more, prevention of autoimmune diseases depends on immune homeostasis, which results from the balances of different T lymphocyte subsets. Therefore, in this study, we have found that urine VTN level was significantly negative associated with %CD19^+^ B cells in PBMC and %CD3^+^CD4-CD8- T cells in the whole blood of inactive JSLE. The similar relationship was found in an analysis for the correlations of VTN with %CD3^+^CD8^+^ suppressor T cells in PBMC of active JSLE. These results imply that due to the higher expression of VTN in active JSLE and lower expression of VTN in inactive JSLE, the corresponding increased %CD3^+^CD8^+^ suppressor T cells in active JSLE and higher %CD19^+^ B cells in inactive JSLE may be involved into the autoinflammation and accumulation of MAC. This reminded that the urine VTN level may become a novel biomarker for the diagnostics of JSLE progression.

Inflammasomes are intracellular multiprotein complexes that coordinate antipathogenic host defense during inflammatory responses in myeloid cells in inflammatory autoimmune rheumatic diseases, especially macrophages [45]. Interesting, VTN has been demonstrated to induce cell pyroptosis through triggering NLRP3 inflammasomes, leading to caspase-1 activation, cleaving GSDMD-N, and IL-18, while no significant difference of ASC expression was obtained in response to VTN stimulation. Further analysis showed that VTN caused a drastic enhancement of phosphorylation of NF-*κ*B, a critical regulator of cell pyroptosis. However, the further work is required to address how VTN regulated NF-*κ*B activity.

In summary, the present study demonstrated the differential abundance of proteins in the urine of JSLE patients, which provided a new insight into the prognosis and development of JSLE. Particularly, the urinary VTN was considered to be the potential biomarkers, which deserved to be further studied before their utility for JSLE diagnosis.

## Figures and Tables

**Table 1:** Demography of clinical characteristics for JSLE patients and healthy controls (mean ± SD).

Parameters	Healthy controls (*n* = 9)	Inactive JSLE (*n* = 9)	Active JSLE (*n* = 10)
Age	3.38 ± 1.02	9.80 ± 1.00^∗^	10.80 ± 0.97^∗^
Gender (M/F)	2/7	0/9	0/10
Disease duration (year)	NA.	1.44 ± 0.69	2.03 ± 1.65
SLEDAI	NA.	4.11 ± 4.08	11.50 ± 7.29^#^
SLICC	NA.	1.56 ± 1.59	3.60 ± 2.59
Hematuria	0/9	1/9	5/10^∗^
Proteinuria	0/9	1/9	4/10
C3 (g/L)	NA.	0.75 ± 0.13	0.38 ± 0.39^#^
C4 (g/L)	NA.	0.14 ± 0.04	0.06 ± 0.05^##^
ANA (g/L)	NA.	16.71 ± 14.85	417.6 ± 201.8^###^
dsDNA (g/L)	NA.	14.52 ± 12.27	443.1 ± 309.7^##^
ESR (mm/h)	NA.	29.11 ± 22.86	6.50 ± 6.76^#^
Creatinine (*μ*mol/L)	22.87 ± 4.37	61.13 ± 15.99^∗^	33.30 ± 1.90
Uric acid (*μ*mol/L)	293.80 ± 107.20	399.40 ± 90.87	318.70 ± 85.48
AST/ALT ratio	2.54 ± 0.79	1.47 ± 0.62^∗∗^	1.36 ± 0.72^∗∗^
CRP (mg/L)	8.94 ± 7.64	5.12 ± 7.18	2.13 ± 3.29
WBC (10^9^/L)	6.70 ± 4.29	7.73 ± 2.19	7 .46 ± 1.78
HB (g/L)	126.10 ± 31.90	116.00 ± 21.33	96.78 ± 25.92^∗^
PLT (g/L)	238.20 ± 99.58	246.70 ± 108.70	260.80 ± 129.20

The statistically significant differences were shown between JSLE patients and healthy controls (^∗^*p* < 0.05, ^∗∗^*p* < 0.01), as well as the significant differences between the active and inactive JSLE patients (^#^*p* < 0.05, ^##^*p* < 0.01, and ^###^*p* < 0.001). There are no statistically significant differences in the parameters of gender, disease duration, SLICC, proteinuria, uric acid, CRP, WBC, and PLT among groups.

## Data Availability

The datasets used and/or analyzed during the current study are available from the corresponding author on reasonable request.

## Abbreviations

ASC: Apoptosis-associated speck-like protein containing a CARD
ALS: Amyotrophic lateral sclerosis
ALT: Alanine amino transferase
ANA: Antinuclear antibody
AST: Aspartate amino transferase
BILAG: British Isles Lupus Assessment Group Index
CIC: Circulating immune complexes
COL18A1: Collagen type XVIII alpha 1
CRP: C-reactive protein
DEGs: Differentially expressed genes
dsDNA: Double-stranded DNA
EPHA4: Ephrin type-A receptor 4
ESR: Erythrocyte sedimentation rate
FBLN5: Fibulin
F11R: Junctional adhesion molecule-A/F11 receptor
GO: Gene Ontology
GSDMD: Gasdermin D
HB: Hemoglobin
IL: Interleukin
KEGG: Kyoto Encyclopedia of Genes and Genomes
LC: Liquid chromatography
MAC: Membrane attack complex
MS: Mass spectrometry
NF-*κ*B: Nuclear factor kappa-light-chain-enhancer of activated B cells
NLRP3: Nod-like receptor family protein 3
PBMC: Peripheral blood mononuclear cell
PLT: Blood platelet
PPI: Protein-protein interaction
PRNP: Prion protein
JSLE: Juvenile systemic lupus erythematosus
SLEDAI: SLE Disease Activity Index
SLICC: SLE International Collaborating Clinics
STRING: Search Tool for the Retrieval of Interacting Genes
S100: S100 calcium binding protein
uPAR: Urokinase plasminogen activator
VTN: Vitronectin
WBC: White blood cell.
